# Effect of Partial Sick Leave on Sick Leave Duration in Employees with Musculoskeletal Disorders

**DOI:** 10.1007/s10926-019-09864-z

**Published:** 2019-10-24

**Authors:** Lisa C. Bosman, Jos W. R. Twisk, Anna S. Geraedts, Martijn W. Heymans

**Affiliations:** 1 Department of Epidemiology and Biostatistics, Amsterdam Public Health Research Institute, Amsterdam UMC, Vrije Universiteit Amsterdam, De Boelelaan 1089a, 1081 HV Amsterdam, The Netherlands; 2 ArboNed Occupational Health Service, Utrecht, The Netherlands

**Keywords:** Musculoskeletal disorders, Occupational health practice, Partial sick leave, Return to work, Sickness absence

## Abstract

*Objective* This study determined if partial sick leave was associated with a shorter duration of sick leave due to musculoskeletal disorders (MSD) based on routinely collected health data in Dutch sick-listed employees. Furthermore, the effect of timing of partial sick leave on sick leave duration was determined. *Methods* This cohort study consisted of 771 employees with partial sick leave and 198 employees with full-time sick leave who participated in an occupational health check, and had sick leave due to MSD for minimally 4 weeks and were diagnosed by an occupational physician. Multivariable linear regression models were performed to determine the effects of partial sick leave (unadjusted and adjusted for confounders and MSD diagnosis) and Kaplan–Meier curves were presented for visualization of return to work for different timings of starting partial sick leave. Furthermore, linear regression analysis were done in subsets of employees with different minimal durations of sick leave to estimate the effects of timing of partial sick leave. *Results* Initial results suggest that partial sick leave was associated with longer sick leave duration, also when adjusted for confounders and sick leave diagnosis. Secondary results which accounted for the timing of partial sick leave suggest that partial sick leave had no effect on the duration of sick leave. *Conclusion* Partial sick leave does not influence MSD sick leave duration in this study when accounting for the timing of partial sick leave.

## Introduction

One of the leading causes of sick leave in European countries is musculoskeletal disorders (MSD) [1]. To prevent employees with MSD of work disability, guidelines advice employees with MSD to stay active, or increase activity and try modified work [2]. This advice agrees with the attitude of employees, as most employees with MSD complaints for more than 6 months who visit an occupational physician for medical consultation consider themselves able to work partially [3]. However, when the duration of symptoms is short, the more likely employees are able to work partially [3]. In the Netherlands and in the Nordic countries of Europe it is possible to have partial sick leave, which means that an employee will work for reduced hours. In the Nordic countries partial sick leave is compensated by partial sickness allowance [4]. Adaptation of job tasks or working hours (partial sick leave) were the most commonly applied interventions among employees sick-listed due to low back pain in a multinational cohort with employees from in Denmark, Germany, Israel, Sweden, the Netherlands, and the United States [5].

Partial sick leave is often implemented in occupational health care, without strong scientific evidence for its positive effect on duration of sick leave. A randomized controlled trail (RCT) studied the effectiveness of partial sick leave on the duration of sick leave due to musculoskeletal pain [6]. They found that employees starting partial sick leave early in their sick leave episode returned to work sooner and had less recurrent sick leave periods. Another RCT study included adapting working hours as part of an intervention, found no significant effect on sick leave duration in employees with low back pain [7]. Although RCTs are seen as the golden standard for determining treatment effects, these RCT’s alone provides insufficient evidence. Carrying out RCT’s on the effects of sick leave are often not considered feasible by general practitioners [8]. Its feasibility is also reduced by legislation in the Netherlands, as employees are required to accept modified duties if this does not affect their medical situation.

Recently, researchers have shown an increased interest in determining the effect of partial sick leave from observational data [9]. Determining the effect of partial sick leave on sick leave duration with observational data faces a lot of challenges, as the recommendation or prescription of partial sick leave depends on multiple factors. In the Netherlands, after 4–6 weeks of sick-listing, an employee visits an occupational physician for an official diagnosis and they will develop a plan of action for return to work. When an occupational physician expects that an employee has a more severe MSD, they might not advise them to work partially if that would aggravate the complaints, because employees are required to accept modified duties if this does not affect their medical situation negatively according to Dutch legislation. Furthermore, the possibility of partial sick leave is also dependent on work and personal characteristics, for example, in a larger company it is more likely that another employee can take over work of the sick-listed employee.

Several studies determined the effect of partial sick leave for employees with only MSD sick leave and showed inconsistent results. One observational study showed that partial sick leave is associated with higher recovery [10]. The effect weakened over a longer time-period, but was still significant in their maximum follow-up period of 6 months. In a study with employees sick-listed minimally 3–4 months due to low back pain, partial sick leave was only associated with shorter sick leave for workers who had a minimal sick leave duration of 200 days. Contrary to the previous study, before this cut-off point, no differences were found [5]. Another study, with employees sick-listed for minimally 2–6 weeks due to MSD without specific pathology, showed no overall effect of partial sick leave. Even more, when timing of partial sick leave was considered, longer durations of sick leave were reported when modified work was started after 7 weeks of full-time sick-listing [11]. However, starting later with partial sick leave is only possible if employees experience longer sick leaves, which might bias this effect. These results indicate that timing of partial sick leave might be important to consider when determining the effect of partial sick leave.

As partial sick leave is often implemented in occupational health care, however with inconsistent findings in previous studies, this study aims to determine if partial sick leave is associated with a shorter duration of sick leave based on routinely collected health data in Dutch sick-listed employees. The secondary aim of this study is to analyze the effect of timing on the effect of partial sick leave on sick leave duration.

## Methods

### Study Design and Population

This study was set up as a cohort study and routinely collected data from an occupational health service was used. In the Netherlands, employers have to offer employees an occupational health check at least once every 4 years. The health checks consists of a questionnaire on work and health, followed by a medical examination if appropriate. The study population consisted of 969 employees who participated in an occupational health check at one of the larges occupational health services in the Netherlands between 2012 and 2017 and reported MSD sick leave with a minimum of 4 weeks within 12 months after their occupational health check. The health checks are assessed in companies across various industries. We did not included employees with < 4 weeks of sick leave, as we would expect that partial sick leave would not be recommended to employees who would return to work on short term. Participation in the occupational health checks were voluntary. Occupational health check participants gave consent for the use of their data for reports on group level, but not at the individual level. Therefore, the personal information of employees are removed from the data files before data analysis to ensure anonymity.

### Outcome Variable

In The Netherlands, sick leaves are certified by an occupational physician (OP) within 6 weeks of reporting sick. OPs certify sick leaves with a diagnostic code related to the 10th International Classification of Diseases (ICD-10). Sick leave OP-certified within the ICD-10 chapter XIII (Diseases of the musculoskeletal system and connective tissue) was defined as MSD sick leave. The outcome of this study was the duration of MSD sick leave and was registered in days. Sick leave duration did not follow a normal distribution, therefore a natural logarithm transformation was made to ensure a normal distribution.

### Partial Sick Leave Variable

Partial sick leave is defined as having worked for reduced hours during sick leave. Sick leave was originally reported in percentages, 0% indicating fully returned to work and 100% fully sick-listed. In this study partial sick leave was dichotomized. Employees who were first 100% sick-listed and in the subsequent modification of sick leave 0% sick-listed, were indicated as having no partial sick leave. All other variations were considered as having partial sick leave.

### Potential Confounders

From the occupational health check individual characteristics, heath-, and work-related factors were obtained, which were potential confounders for partial sick leave and sick leave duration according to the literature [5, 6, 10–14].

*Individual characteristics* included gender, age, and educational level, which was measured on a seven grade scale and was categorised in low (none, primary education and junior vocational education), medium (secondary general education and senior vocational education) and high (higher professional education and university) education.

*Health*-*related factors* included body mass index (BMI), smoking (yes or no), and complaints of musculoskeletal disorders, which included pain/stiffness in the back, arms/neck/shoulders, hand/wrist, and hip/knee/ankle/foot. For each type of complaint response options ranged from 1 “not” to 4 “most of the time” and were averaged resulting in a range of 1–4.

*Work*-*related factors* included company size (< 500 employees or more than 500 employees), actual working hours reported by the employees, and commute time. Work pace and social support were assessed by the Dutch questionnaire QEEW [15]. Work pace included 5 items, with the response options ranging from 1 “not/never” to 5 “always”; responses were averaged, resulting in a scale score ranging 1–5. Social support consisted of three scales: social support by supervisor, colleagues, and by family/friends. Each scale consisted of three items with response options ranging from 1 “no” to 5 “very often or always” and were averaged to scores in a range 1–5. The three scales were averaged to form one social support scale. Physical job demands were assessed with 7 items, such as “Do you lift, push, pull or carry heavy weights (more than 23 kilograms)?”, or “Do you work in uncomfortable postures?”. Response options ranged from 1 “no/never” to 4 “most of the time” and were averaged resulting in a scale score between 1 and 4.

*Sick leave*-*related* factors were determined from sick leave registers from the occupational health service and included sick leave history (yes or no), sick leave diagnosis, which were categorized in complaints (such as back or neck pain), injuries/fractures (such as fracture of one leg, or amputation of fingers), and chronic musculoskeletal disorders (such as arthritis or repetitive strain injury). Furthermore timing of starting partial sick leave was assessed and categorized in no partial sick leave, onset 0–42 days, onset 43–60 days, onset 61–90 days, onset 90–180 days, and onset > 180 days.

### Statistical Analysis

The effects of partial sick leave on sick leave duration were determined with linear regression analyses with a natural logarithm transformation of the outcome sick leave duration. Adjustments were made for all confounders and in an additional analysis also for sick leave diagnosis. To determine whether the effects differed in each diagnosis group, an interaction term was included between the partial sick leave variable and diagnosis group. To visualize the effect of timing of partial sick leave on the duration of sick leave, Kaplan–Meier curves were produced for each category of timing. To take into account that starting partial sick leave later on in the sick leave period is inherent to longer sick leave duration, subsets were created in the dataset with employees with a minimum of 42, 60, 90 and 180 days of sick leave duration corresponding to the timing categories. In these subsets the effect of partial sick leave on sick leave duration was determined.

To deal with the missing values in the variables, multiple imputation was applied by using the multivariate imputation by chained equations (MICE) package in R statistical software [16]. According to White et al. [17] the number of imputations can be derived from the percentage of incomplete cases. Percentages of missing values per variable ranged from 0 to 34.5%. When using the selected confounder variables and treatment variable, the dataset contained 46% incomplete cases, therefore 46 different imputed datasets were created. Regression analyses adjusted for confounders were performed on the imputed data and estimates and corresponding standard error (SE) were pooled.

## Results

969 employees had been on sick leave for a minimum of 4 weeks and were diagnosed with MSD sick leave by an occupational physician. Of these employees, the majority had partial sick leave (79.6%) (Table 1). Furthermore, every employee in this study population returned to work.

**Table 1:** Characteristics of study population with and without partial sick leave

	With partial sick leave (n = 771)		Without partial sick leave (n = 198)	
		Missing %		Missing %
Gender [n (%)]		0		0
Men	609 (79.0)		159 (80.3)	
Women	162 (21.0)		39 (19.7)	
Age, mean (sd)	48.7 (10.5)	0.001	49.3 (10.3)	0
Educational level [n (%)]		11 (1.4)		0
Low	242 (31.4)		66 (33.3)	
Medium	398 (51.6)		105 (53.0)	
High	120 (15.6)		27 (13.6)	
BMI, mean (sd)	26.8 (4.2)	7.1	27.2 (4.6)	7.0
Smoking [n (%)]		3.9		3.0
Yes	198 (25.7)		52 (26.3)	
No	543 (70.4)		140 (70.7)	
Complaints of MSD [mean (sd)]	2.0 (0.6)	19.6	2.0 (0.6)	16.7
Company size [n (%)]		0		0
Less than 500 employees	272 (35.3)		72 (36.4)	
More than 500 employees	499 (64.7)		126 (63.6)	
Working hours, mean (sd)	36.3 (7.3)	1.7	36.9 (7.9)	5.1
Work pace, mean (sd)	2.6 (0.8)	1.9	2.6 (0.8)	6.5
Social support, mean (sd)	3.6 (0.7)	5.7	3.7 (0.7)	10.0
Commute time, mean (sd)	54.4 (41.8)	1.9	58.3 (50.2)	5.1
Physical job demands, mean (sd)	2.1 (0.5)	34.5	2.0 (0.5)	27.2
Sick leave history, n (%)		11.0		7.1
Yes	579 (75.1)		148 (74.7)	
No	107 (13.9)		36 (18.2)	
Sick leave diagnosis [n (%)]		0		0
Complaints of MSD	253 (32.8)		65 (32.8)	
Fractures/injuries	235 (30.5)		62 (31.3)	
Chronic MSD	283 (36.7)		71 (35.9)	
Timing partial sick leave (days), mean (sd)	51.8 (59.4)	0	–	–
Sick leave duration, mean (sd)	135.3 (123.9)	0	102.1 (120.5)	0

The unadjusted effect of partial sick leave on sick leave duration was 1.40, which indicates that employees with partial sick leave had 1.40 times longer sick leave than the employees with fulltime sick leave (Table 2). When adjusted for confounders, this effect changed to 1.41 and to 1.39 when adjusted for sick leave diagnosis. No significant effect modification was found by sick leave diagnosis.

**Table 2:** Unadjusted and adjusted effects of partial sick leave on sick leave duration

	Exp(B)	95% CI	p
Partial sick leave, unadjusted	1.40	1.39–1.40	< 0.01
Partial sick leave, adjusted for confounders	1.41	1.41–1.42	< 0.01
Partial sick leave, adjusted for sick leave diagnosis	1.39	1.39–1.40	< 0.01

**Fig. 1:**
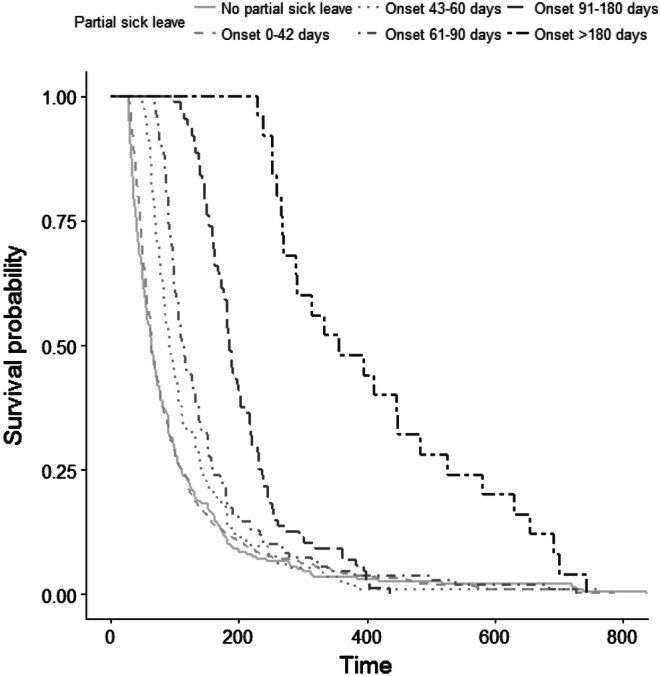
Survival curve for sick leave duration among employees with different timing of starting partial sick leave

Figure 1 shows the survival curves for the different timings of starting partial sick leave. Especially the employees who started partial sick leave after 180 days had a significant longer sick leave duration. However, this is also inherent to creating these groups, as they have a sick leave duration with a minimum of 180 days.

To account for the possible bias of timing of sick leave, the effects of timing of starting partial sick leave are determined in subsets of employees with different minimal duration of sick leave in Table 3. In the first subset of employees who are sick-listed for minimally 42 days, no significant difference was found in sick leave duration between starting partial sick leave between 0 and 42 days and no partial sick leave. In the second subset, both starting partial sick leave between 0 and 42 days, and between 43 and 60 days did not result in a significantly longer or shorter sick leave duration in comparison with no partial sick leave. In the third subset, starting partial sick leave between 0 and 90 days show no significant difference in sick leave duration compared to no partial sick leave. In the last subset, starting partial sick leave between 0 and 90 days does not result in a difference in sick leave duration, compared to no partial sick leave. However, in this subset, starting partial sick leave between 91 and 180 days results in a significant lower sick leave duration. The employees who start partial sick leave between 90 days and 180 days have 0.75 times shorter sick leave duration.

**Table 3:** Effects of timing of partial sick leave on duration of sick leave in subsets with a minimum duration of sick leave

	Exp(B)	95% CI	P
Employees with min. 42 days sick leave (N = 837)			
Onset 0–42 days	0.93	0.83–1.05	0.24
Employees with min. 60 days sick leave (N = 680)			
Onset 0–42 days	0.97	0.86–1.10	0.66
Onset 43–60 days	0.91	0.79–1.06	0.22
Employees with min. 90 days sick leave (N = 473)			
Onset 0–42 days	1.03	0.89–1.18	0.70
Onset 43–60 days	0.91	0.77–1.08	0.28
Onset 61–90 days	1.13	0.76–1.03	0.16
Employees with min. 180 days sick leave (N = 338)			
Onset 0–42 days	1.00	0.82–1.21	0.99
Onset 43–60 days	0.81	0.64–1.03	0.08
Onset 61–90 days	0.90	0.71–1.27	0.35
Onset 91–180 days	0.75	0.62–0.91	< 0.01

Reference category = no partial sick leave

## Discussion

The objective of this study was to determine the effect of partial sick leave on the duration of sick leave. The results show that partial sick leave was associated with longer sick leave duration. However, when the timing of partial sick leave was taken into account, the negative effect of partial sick leave disappears and no difference in sick leave duration is found due to partial sick leave. Even more, a positive effect of partial sick leave was found in employees who started partial sick leave between 91 and 180 days.

Comparing the results of our study to other studies should be done with caution, as multiple factors differ between the studies which could have impacted the results. In agreement with our results, several studies found no effect of partial sick leave or modified work on sick leave duration [5, 7, 11, 18]. Furthermore, it was reported that in the group of employees who are sick-listed for minimally 200 days, partial sick leave was associated with a higher rate of return to work [5]. In our study we found, in employees who were sick-listed for minimally 180 days, that starting partial sick leave between 61 and 90 days was associated with a reduced sick leave duration. However, other starting points of partial sick leave did not result in significantly different sick leave duration from no partial sick leave. Likewise, the results of van Duijin et al. [11] are also partially in line with our results. They showed no overall effect of modified work on time to return to work, but when modified work was started after 7 weeks or more, sick leave duration significantly increased, according to their Kaplan–Meier curves. Our study showed the same results when analyzed in the same manner. However, they did not take into account that when starting later with partial sick leave, sick leave duration inherently increases. Furthermore, modified work in the study of van Duijin et al. [11] included both reduced work hours and alternative work tasks, whereas our study only looked at adaptation of working hours. This might have increased the effect of modified work on sick leave in van Duijin et al. [11], as in a previous study an overall effect was found for adaptation of job task, but not for adaptation of working hours [5].

Our results were in contrast with the results of several other studies, which reported positive effects of partial sick leave with regards to return to work and recurrent sick leaves [6, 10, 19–21]. However, in an RCT no positive effect of partial sick leave was found for time to return to work in one large participating organization, which contributed one-third of the study population. No explanations for this were provided from subgroup analysis [6]. In one of the observational study instrumental variable analysis (IV) was used to estimate the effect of partial sick leave on return to work [10]. Their crude analysis indicated that partial sick leave was associated with a lower probability of recovery. As an explanation for this reversed effect after IV analysis, they argue that employees who had partial sick leave were female and older, which were characteristics associated with a lower recovery in their study and caused bias in the crude analysis [10]. However, in our study gender and age were not confounders in the association between partial sick leave and sick leave duration and adjusting for these confounders did not result in a different effect. Furthermore, the study of Turner et al. [19] also considered the timing of modified work in the determination of the effect of modified work on work disability. They reported that starting modified work within 3 weeks of sick-listing predicts a lower probability of work disability. This is contradictive to our study, as starting partial sick leave between 0 and 42 days did not result in a significantly shorter sick leave duration. Also starting partial sick leave between 0 and 28 days was not associated with shorter sick leave duration (data not shown in manuscript). In agreement with our results, an RCT study found that an early intervention with adaptation of working hours did not result in significant reduced sick leave duration [7]. An important difference with the study of Turner et al. [19] and our study is that they included employees sick-listed for minimally 4 days, whereas in our study only employees were included who were sick-listed for minimally 4 weeks. We choose to include only employees with a minimum of 4 weeks, as in the Netherlands employees visit an occupational physician after 4–6 weeks of sick-listing. At that time a diagnosis is confirmed and the occupational physician can advise on partial sick leave.

In our study, a very high percentage of partial sick leave was reported (80%), whereas in the study of van Duijin et al. [11] half of this percentage had partial sick leave (40%). Anema et al. [5] reported that 46% of the employees had partial sick leave, and in the study of Andrén and Svensson [10] only 12% of the employees started with partial sick leave. All employees in our study population had to adhere to legislation on accepting adaptation of work when considered appropriate by an occupational physician, which explains the high prevalence of partial sick leave. In the study of van Duijin et al. [11], which was also a Dutch study, this law was enforced during their study, therefore only part of their study population had to adhere to it. This difference in prevalence of partial sick leave could have influenced the effect of partial sick leave on sick leave duration, as in our study some employees with partial sick leave would not have gotten the advice for partial sick leave before the new law was enforced. This difference in legislation makes comparison with other studies more difficult.

### Why No Association with Partial Sick Leave was Found

Given the inconsistency of our results with results from other studies [5, 6, 10, 11], it is important to consider whether our study appropriately adjusted for potential confounders. In our study none of the confounders showed to influence the effect of partial sick leave. Possibly we did not measure all relevant confounders, for example disability due to the complaints [11], type of occupation [10], or whether also adaptation of job tasks were present [5]. Whether an employee will have partial sick leave dependents on a variety of factors, however none of the measured confounders seemed to provide significant information on this. For example, contrary to our results, in a previous study high physical demands were associated with not being assigned to modified work [11]. This can be considered a unexpected finding, as physical demands is a known risk factor for low back pain and related sick leave [22, 23]. Therefore it can be expected that working partially and reducing the physical demands at work is advised for these employees. Contrary, in jobs with high physical demands less opportunities may exist to work partially or perform modified work. It can therefore be suggested that these two possibilities even each other out, explaining why no associated with partial sick leave is found in our study.

It can also be expected that the severity of sick leave diagnosis influences the effect of partial sick leave on sick leave duration. This study tried to gain insight into this by examining the three groups of MSD diagnosis: complaints, fractures/injuries, and chronic MSD. This categorization might be related to severity, however the diagnosis groups did not influence the effect of partial sick leave on sick leave duration. When unmeasured confounding is present, IV analysis is considered a good method to account for this when a strong and valid IV is present in the data. This method was used in the study of Andrén and Svensson [10], however they also indicate that their IV’s were fairly weak, which could have let to bias in the estimated effect. The IV method was not applicable in our study, as a strong IV was not available in our dataset.

### Strengths and Limitations

An important strength of the present study is that we accounted for the timing of partial sick leave in the effect of partial sick leave on sick leave. Another strength of this study was the use of reported sick leave data as it minimized self-report bias [24]. We used data collected by an occupational health service with various economic sectors, therefore our population was also heterogeneous, which results in higher generalizability of our results.

In addition to possible unmeasured confounding, another limitation of this study is the amount of missing data. The cause of the missing values in the confounders lies in the variation of content of the occupational health check among the different companies. Some companies had more elaborate health check questionnaires than others. We dealt with this limitation by using multiple imputation in the confounders and pooling regression coefficients afterwards. Thus, incomplete cases still provided information for the regression models.

Finally, the data available for this study was limited. We used information from occupational health checks and combined this with sick leave data with a follow-up of 12 months. This is not an optimal setting, as some of the confounding variables might have changed over time between the assessment of the occupational health check and the sick leave episode, such as work pressure or social support. Ideally, one would have taken questionnaires including confounders measured at the start of sick leave. Furthermore no information was available on adjustment of work tasks, only on reduced work hours. Sick leave duration in low back pain patients could also be reduced by accommodating adjustment of work tasks, such as restricting lifting weights. It was possible that some of the employees on partial sick leave also had adjustment of work tasks, which could have influenced the results, as in previous studies a positive effect was found for adjustment of work tasks on sick leave [5, 11]. Also no information was available on received treatments or interventions, which could also have influenced the results. The type of occupation of the employees was not available in this study, however physical demands at work and the level of education may provide some information on the type of occupation. These two variables were not found to be relevant confounders in the effect of partial sick leave on sick leave duration.

This study also lacked in information on comorbidity, MSD related or not, as this increases risks for adverse work outcomes [25]. Another limitation is that we simplified partial sick leave by dichotomizing it, although some employees could have worked more than others during partial sick leave, which might influence the results. Lastly, a limitation to consider is that the employees in our study population were sick-listed for minimally 4 weeks. Therefore the effect of partial sick leave in short sick leaves cannot be estimated from this study. Previous studies indicate that early multi-faceted interventions are effective in reducing sick leave [26, 27]. However, as the Dutch sick leave policies describe that an employee will visit an occupational physician between 4 and 6 weeks, our study can aid the occupational physician in their recommendations on partial sick leave.

### Practical Implications

Although no significant differences were found in sick leave duration when the timing of partial sick leave was accounted for, this could still be in favor of recommending partial sick leave. If the sick leave duration is not effected, work productivity can be gained if employees work partially, which could also relieve costs due to full sick leaves. Furthermore, when it is expected that an employee will be sick-listed for minimally 180 days, a significant reduction in sick leave duration can be expected when partial sick leave is started between 91 and 180 days. Occupational physicians that estimate the duration of sick leave should therefore consider this cut point in their advice for partial sick leave.
